# Pennsylvania State Medical Society

**Published:** 1851-06

**Authors:** 


### PENNSYLVANIA STATE MEDICAL SOCIETY.

The Annual Meeting of this body, composed of delegates from all the County Medical Societies in the State, was held in Philadelphia on Wednesday morning, 28th May, at eleven o'clock, in the Hall of the American Philosophical Society, south Fifth street, below Chestnut; the President, Dr. Wilmer Worthington, of Chester County, being in the chair. Drs. Patterson and Kerfoot, the Secretaries of the Society, having handed in their resignations, Drs. Remington and Morris were appointed to fill the vacancies. On motion of Dr. Jewell, Surgeons of the Army and Navy, and physicians of other States, now in the city, were invited to be present at the sittings of the Society.

The hours of sitting were fixed as follows: From 10 o'clock, A. M., to 2, P. M., and 5 to 7, P. M.

The following committee was appointed to nominate officers for the ensuing year:—Dr. Kerfoot, of Lancaster Co.; Dr. I. R. Walker, of

Chester; Dr. John Bascomb, of Mercer; Dr. J. H. Case, of Perry; Dr. C. D. Gloninger, of Lebanon; Dr. Elwood Harvey, of Delaware; Dr. Peter Nagle, of Berks; Dr. R. E. James, of Northampton; Dr. Joseph B. Ard, of Mifflin; Dr. James H. Stuart, of Erie; Dr. J. M. Gemmill, of Huntingdon; Dr. J. B. Rose, of Blair; Dr. E. Chichester, of Schuylkill; Dr. C. S. Baker, of Bucks; Dr. T. F. Betton, of Philadelphia county; Dr. H. Corson, of Montgomery; and Dr. James W. Kerr, of York. The Society adjourned at 2 o'clock, and reassembled at 5 P. M.

During the afternoon session, Dr. Hays presented reports from various county Medical Societies throughout the State, conveying information which was called for by the action of the State Society. The reports from Berks, Huntingdon and Blair counties embodied interesting accounts of the medical topography of these counties. The report from Blair also contained a variety of statistics of that county, as well as a list of the members of the County Society. The report states that there are eleven regular and thirteen irregular practitioners in that county. The Chester county report gave a history of the County Medical Society, and stated the number of members at forty. The Erie report complained of the opposition to the Society manifested in the county by several physicians of standing, and of the prejudice they had excited in the public mind against the movement. The Mercer report stated the number of members of the County Society at 22, and added that the number of irregular practitioners in the county was much larger, including homœopathists, hydropathists, Indian doctors, &c. The Huntingdon County Society stated in their report that there are 34 regular physicians in that county, besides 4 Thompsonians, and one homœopathist. In Northampton there are also 37 regular physicians, but there are 17 irregulars, of whom 9 are homœopathists. In Berks county there are about 68 practitioners of all kinds, 40 being regulars, and from 28 to 32 irregulars. The Delaware County Society reports a list of its members.

Dr. Isaac Parrish presented an interesting report on epidemics, embodying some valuable information obtained from Philadelphia and the adjacent counties, the reading of which was postponed.

Dr. Condie, from the committee on the subject, reported that a bill for the registration of births, marriages, and deaths, was passed by the Legislature at its last session, but that it had not yet received the signature of the Governor. In view of the latter fact, the report urged the passage of a resolution requesting the Governor to sign the bill. A motion being made that this resolution be passed, quite an animated debate sprang up relative to the merits of the bill.

Dr. Jewell stated that he had been informed that the Governor objected to some of the provisions of the bill, and that he would not sign it, inasmuch as those provisions would render it a dead letter upon the statute book. Dr. C. W. Parrish, of Chester county, strongly objected to the bill on the ground that its provisions imposed an onerous burthen upon the physicians who resided in rural districts, and one which it would be impossible to fulfil according to the strict letter of the law. Dr. Parrish also argued that the phraseology of the bill was loose, untechnical, and inefficient, and said that he was glad to hear that the Governor would not sign the bill.

Dr. Condie, in defending the bill, said that, although this bill is objectionable, yet it ought to be accepted, because it would be very difficult indeed to procure the passage of another. He said that the entire project of registration had encountered the opposition of many physicians in western Pennsylvania, on the ground that it was unnecessary, and calculated to excite prejudice against the medical profession. The Doctor further stated that in the lower house of the Legislature, on a motion to refer the bill to a committee, every physician who was a member voted against it. Dr. Parrish, of Chester, stated that many gentlemen of that county were strongly opposed to the bill, and purposed getting up an agitation for its repeal if it received the Governor's signature. Pending the discussion, the Convention adjourned.

The Society decided to meet for the future in Sansom St. Hall, where ample accommodations were found for the delegates and the large auditory that was in attendance.

*Thursday, May 29th.*—The Society re-assembled at the Sansom street Hall.

The discussion on the subject of the registration of births, marriages and deaths was resumed, the question being on the passage of Dr. Condie's resolution. Dr. Atlee, of Lancaster, defended the bill, and urged that its signature should be favored; because, if it were incomplete, it could afterwards be amended. He said that he had no doubt that, if the Governor did sign the bill, it would be used as a ground of violent political opposition in the impending campaign. He added, that the Governor was an intelligent and liberal minded man, and he relied upon him to do justice to the subject after the canvass was finished.

Dr. Burroughs, of Lancaster, said that he had been opposed to the bill on account of its imperfections; but this opinion had been shaken by conversation with some of its friends. He, however, was still of opinion that if the bill, in its present imperfect state, received the signature of the Governor, under the recommendation of this Society, the latter fact would be used as an argument against its emendation, and would render its amendment impossible. The Doctor also urged that some of the provisions of the bill were too inquisitorial into family secrets.

Dr. C. W. Parrish, of Chester Co., offered the following substitute for the resolution, which was accepted by Dr. Condie, the mover of the original resolution:

*Resolved*, That we hail this first attempt in the State of Pennsylvania to accomplish so important a measure as a token for good; and, though the bill passed by the Legislature, in some of its details, does not fully meet the views and expectations of this Society, still it is earnestly recommended to all upon whom the duty of carrying out its requirements devolves, to comply promptly with all those requirements, as accurately as possible, in case the bill should become a law.

Dr. Emerson defended the provisions of the bill, and asked the objectors to be more specific in stating its defects. He also proceeded to show the value of the statistics which would be obtained by this law, and, in illustration, quoted some very interesting deductions from the same kind of statistics in England and in Massachusetts.

Dr. Baskin, of Mercer county, argued in favor of the bill, as it was the best that could be got; and although it might possibly bear somewhat heavily on the rural physicians, yet they should sacrifice some little convenience in order to obtain the valuable information which would result.

The substitute, as offered above, passed without dissent.

Dr. Baskin then offered a resolution for the appointment of a committee to convey to the Governor the wishes of the society, and its action upon the subject, and that he be asked to sign it.

Dr. Atlee opposed the resolution on the ground that it was indelicate and improper, after the resolution which has already been passed.

A motion was made to amend it by making it the duty of the Secretaries to inform the Governor on the subject.

Dr. Jewell moved that the whole resolution and amendment be indefinitely postponed. Lost—ayes 23, noes 24.

The amendment was accepted.

A motion was made that the resolution be laid on the table. Lost—ayes 21, noes 26.

The resolution, as amended, was then adopted.

On motion of Dr. Emerson, the thanks of the Society were returned by resolution to Edward Armstrong, Esq., of this city, for his active and efficient exertions in procuring the passage of this bill.

Dr. Hays, from a committee on the subject, presented a report from Philadelphia, giving the following statement of the number of physicians in the city and county:—Regular physicians, 397; homœopathic, 42; hydropathic, 2; Thompsonian, 50; advertising doctors, 32; druggist physicians, 37; nondescripts, 42; total, 582. It is further stated that there are probably some omissions which would raise the total to 600.

The report of the committee on epidemics, offered by Dr. Parrish, of this city, was now taken up, and the accompanying county reports read seriatim. These reports were from Philadelphia, Delaware, Montgomery, Chester, Berks, Huntingdon and Blair. The report of the committee expressed their regret that so few county societies have reported on the subject, and they recommend that, as this is the first year of the attempt to collect such information, the county societies be requested to continue their committees from year to year, to collect information on the prevailing diseases of the several counties within the year 1851, with a particular description of the origin and progress of any epidemic or contagious disorder which may have occurred within that time, together with any other matters of interest affecting the mortality or health of their respective districts.

The report from Philadelphia was composed of distinct documents, giving the prevailing diseases, general health, natural formation of the soil, character of the buildings, and other matters affecting the sanitary condition of the city proper, Spring Garden and Penn districts, Southwark, Northern Liberties, West Philadelphia, Richmond, Frankford, Byberry, Germantown, etc. All of these reports contained many interesting and important facts relative to the health of Philadelphia.

A motion was made and adopted that a committee of publication, to consist of three members, should be appointed. The Chair appointed Drs. Isaac Parrish, D. F. Condie, and G. W. Norris.

The sanitary report from Delaware County showed that there is in that county one regular physician for every thousand inhabitants, and for every six and a fraction square miles of territory.

We append the following abstract of the

### *Sanitary Report of the County of Philadelphia.*

The diseases of the county of Philadelphia, in their origin and progress, and in the type which they assume, vary according to locality and surroundiug circumstances. In the compactly built portions of the city, disorders show themselves which are rarely seen in the surrounding suburbs, while the rural districts are visited with diseases which never originate in the heart of the city. On this account it is necessary, in considering the diseases of the whole county, to separate the city from the parts which surround it and to view the prevailing diseases in connection with those local circumstances which are known to influence them. With this object, the Philadelphia County Medical Society appointed a committee, composed of gentlemen residing in the several districts, through whom the information contained in this report was obtained. The tables of mortality indicate the deaths from various diseases in the city proper and districts, the boroughs and townships not being included. These tables were carefully prepared at the office of the Board of Health, at the instance of Dr. Wilson Jewell, a member of the committee, and formerly one of that Board. They may be relied on as accurate, so far as the returns made to the Health Office are full and reliable.

The city proper enjoys an excellent reputation for health. It is entirely clear of malarial diseases, and of late years has beea generally exempt from any yellow fever or other malignant epidemic fevers. The cholera of 1832 and 1849 was much less extensive and mortal than in New York and some of the cities of the South and West. Epidemic typhus has not of late years appeared, and the cases of this disease in newly arrived emigrants, or amongst the lowest class of population, which occur more or less every year, rarely spread to any extent.

Infantile diseases, believed to be traceable to our peculiar climate and to confined air in hot weather, are unhappily prevalent during the heat of summer, and sometimes to an alarming extent. This arises in part from the oppressive heat of the city not being relieved by a sea breeze, and from the system of building up confined courts and blind alleys, in which are crowded a large number of families, who are unable to avail themselves of the advantages of country air, when their children are attacked by these destructive complaints.

Philadelphia, in common with many other cities and districts of the United States, has, within the past year, suffered with epidemics of scarlet fever, varioloid, small pox, erysipelas, puerperal fever, and other less grave complaints, which observe certain periodical cycles, the peculiar laws of which are not clearly understood. Our business is with the year 1850, just passed, and we will endeavor briefly to note the diseases which have prevailed during that period.

In addition to the ordinary diseases of the winter months, such as catarrh, pneumonia, pleurisy, &c., the city was visited by an epidemic of scarlet fever, which prevailed, to a considerable extent, from the commencement of the year until the approach of warm weather, when it subsided, and then reappeared in the autumn, continuing through the winter, with various alternations of mildness and severity. In some families this complaint appeared to manifest peculiar malignity, carrying off several children in rapid succession, and in those who escaped with life, leaving behind it marks of its ravages, in protracted and painful secondary symptoms, which sometimes inflicted permanent injury upon the system. The causes of this variety in the intensity of the disease in individuals exposed to the same poison, is quite inexplicable, though the fact is well known.

In several instances under the care of the writer of the report, the disease occurred in adults, and in two of these deaths took place. One of these was a man about 50 years of age, who had not, as far as could be ascertained, been exposed to the disease, and who died on the third day, without communicating the disease to any member of his family, which was composed of several unprotected persons who waited on him during his sickness.

Varicella was prevalent in the early months of the year 1850, and in one instance under the observation of the writer, the eruption of scarlet fever and the vesicles of varicella existed at the same time in the same patient.

Measles also prevailed as an epidemic in the latter part of the winter, and during the spring, declining towards summer. The cases were generally manageable, although a considerable mortality from this disease is reported.

Pertussis was quite prevalent during the spring and summer, and many cases continued on through autumn. The disease was sometimes severe, and when complicated with dentition or pneumonia, was often fatal. It appears from the mortuary tables of the Board of Health that 24 deaths occurred from this disease in the eighth month, (August) and only one death in the first, month(January.) This fact would seem to militate against the opinion that whooping cough is commonly fatal from the complication of inflammatory affections of the lungs; while it favors the idea that death in this disease more frequently happens from nervous exhaustion, as the latter is powerfully promoted by intense heat. An important practical suggestion may be derived from this fact, viz. that an early use of tonics, particularly of bark, with nutritious diet and free exercise in the open air, is very important to counteract the tendency to exhaustion: while depletion and nauseating medicines to relieve fancied inflammation should be carefully avoided.

In the early part of the year a few cases of erysipelas were observed, though generally of a mild, non-malignant type, and although affecting the face and scalp, rarely producing death. The same disease appeared again in the autumn, and continued during the winter.

*Typhus Fever.*—Dr. Lawrence Turnbull writes to the Committee that in February, 1850, this disease appeared as an epidemic in the southern sections of the city. Though severe, none of the cases were fatal. Medicines appeared to exert but little influence over the course and duration of the disease. A sustaining treatment proved more ameliorating than a depleting one.

During the summer months of 1850, the usual forms of bowel disorders, viz : cholera morbus, cholera infantum, and dysentery, made their appearance. Of this last named disease there was an unusual prevalence within the city limits, though it was not sufficiently marked to be considered as an epidemic. Until within the past eight or ten years, dysentery, at least in its severe forms, was comparatively a rare disease in the closely built portions of Philadelphia; and where severe cases did occur, they could usually be traced to the country, thus confirming the opinion expressed by many writers, that this disease owes its origin to exhalations from stagnant pools, and from wet and marshy grounds, and is traceable to the same causes which produce intermittent, and remittent fevers. Certain it is that the epidemic dysentery, which prevails at certain seasons as a fatal and destructive disease in rural districts, was rarely seen within the limits of the city proper until within a few years past, and even now it may be questionable how far the cases which prove dangerous or fatal within these limits, partake of the character of the epidemic form. 413 deaths from dysentery are reported by the Board of Health within the year, although we have no means of determining how many of these were in the city proper, and how many in the suburban districts, which are known to be more or less subject to malarial influences. The larger number of these deaths were in young subjects, and it is probable many of these occurred in children during the period of dentition, and might have been classed by some practitioners under the head of cholera infantum. But making allowance for these abatements, the number of fatal cases of this disorder is unusually large. From the years 1836 to 1847, inclusive, the greatest mortality from dysentery in the city and districts in any one year was in 1847, being 160; and the smallest in 1844, being 47. In 1848 the mortality ran up to 304. The tables for 1849 are not accessible, but in 1850, we find it at 413, the highest rate for 14 years, making all due allowances for the increase of population. The causes of this increased prevalence of dysentery within the city limits are worthy of investigation, whether they be of local influence or form one of those periodical cycles which are known to govern many diseases.

Cholera infantum prevailed to the usual extent in 1850, and with results varying but little from former years. The mortality in 1847 was 442, and in 1848 it was 457, being but 46 less than in 1850.

During the latter part of summer, and until the occurrence of frost, intermittent and remittent fevers prevail, more or less, in the suburban districts, and a few cases find their way into the city proper. In the year 1850, so far as our observation enables us to speak, the cases were fewer than usual, both in the city and in the surrounding country.

A considerable number of cases of typhoid fever occurred during the same period, some of which originated in malarial districts, and seemed in the early symptoms, more allied to ordinary remittent than to the peculiar enteric fever described by modern writers under the term typhoid. The total number of deaths from typhoid fever in 1850, reported to the Board of Health, is 107; the highest number, 27, occurring in the 10th month, (October.) How many of these cases originated in the city proper it is impossible to say; while in the present state of opinion on this subject it is very difficult to arrive at accurate results in regard to its progress. What is denominated by some practitioners typhus, is called by others typhoid, while remittent or even intermittent fevers of a protracted and low type may be classed as typhoid by some observers. Personal observations have convinced us that the peculiar form of fever described by Louis, Bartlett, and others, as typhoid, and by Dr. Wood as enteric, has existed within the city limits during the year; and the testimony of medical friends well qualified to judge, is equally clear upon this point.

During the latter months of the year 1850, we had a repetition of the diseases of the previous winter, viz.: scarlet fever, measles, whooping cough, erysipelas, &c. There was also an extraordinary prevalence of abscesses in the form of boils, occurring in persons not subject to such complaints, with many cases of anthrax, and of paronychia. At a meeting of the County Medical Society, there was such a remarkable concurrence of sentiment amongst the physicians present as to the frequency of these cases in their practice, as to induce the belief that some peculiar state of the atmosphere or epidemic influence was operating to produce it. It was remarked that the cases of paronychia were most numerous amongst house maids and laboring people, and some of them were observed to be very tedious in healing, even after free opening made in the early stage of the inflammation. The tendency to this form of the disease continues to the present time, though in a less marked degree.

*Table of Deaths in Philadelphia in* 1850 *from epidemic, endemic and contagious diseases.*

| | Males. | Females. | Boys. | Girls. | Totals. |
|---|---|---|---|---|---|
| Cholera Infantum, - - | 274 | 229 | 214 | 229 | 503 |
| " Morbus, - - | 16 | 8 | 3 | 3 | 24 |
| Diarrhœa, - - - | 104 | 104 | 77 | 69 | 208 |
| Dysentery, - - - | 241 | 172 | 134 | 88 | 413 |
| Erysipelas, - - - | 25 | 29 | 11 | 22 | 54 |
| Fever, Intermittent, - - | 12 | 4 | 8 | 2 | 16 |
| " Remittent, - - | 24 | 26 | 7 | 14 | 50 |
| " Typhus, - - - | 46 | 29 | 5 | 9 | 75 |
| " Typhoid, - - | 70 | 37 | 20 | 14 | 107 |
| " Scarlet, - - - | 232 | 207 | 262 | 197 | 439 |
| Measles, - - - - | 40 | 30 | 40 | 30 | 70 |
| Small Pox, - - - | 26 | 14 | 19 | 12 | 40 |
| Whooping Cough, - - | 62 | 52 | 62 | 52 | 114 |
| Totals. | 1172 | 941 | 892 | 741 | 2113 |

The monthly mortality, in 1850, was as follows: January 603, February 579,

March 580, April 596, May 792, June 708, July 1041, August 1150, September 647, October 701, November 515, December 558; aggregate 8510. The mortality in the first year of life is nearly 50 per cent. of the whole number of deaths under 20 years of age, and those deaths which occurred within the 5th year, constitute over five-sixths of those under 20 years of age. The proportional infantile mortality, according to months, stands thus: August 807, July 707, June 491, May 470, September 382, April 378, October 365, March 361, February 355, January 347, December 305, November 300.

The Report from the City of Philadelphia is by Dr. Isaac Parrish.

Of the Reports from the various Districts of the County, we make the following abstracts:

Northern Liberties.—This report is from Dr. Thomas H. Yardley. "The Schuylkill water is furnished here to 5300 houses, 720 of which are provided with baths: in addition to which there is a select bathing establishment for respectable females, furnished with hot, cold and shower baths. It is managed by a committee of ladies, who are furnished with the funds to carry it on by voluntary contributions, and who, on being satisfied of the respectability of the applicant, gives her a ticket, which is not transferable, but admits her during the bathing season. It has been in operation two years; and during the first year 1000 baths were taken. It is highly appreciated, and has been of invaluable service to the health and comfort of the class of individuals for whom it was established."

The gas manufactory in the district of the Northern Liberties has greatly improved the health of the neighborhood in which it is situated, which was the lowest and most unhealthy part of the District. The residents there have previously been usually subject to dysentery and autumnal fevers, and during the cholera season of 1833, previous to the erection of the gas works, the disease was more prevalent and fatal here than in any other part of the district. During the last epidemic not a case of cholera occurred in the neighborhood, and dysentery and autumnal fevers have entirely disappeared. The superintendent further states that several persons affected with symptoms of a pulmonary complaint have been employed at the gas works, and have become perfectly well and hearty men.

Richmond.—This district is exposed to malarial poison from an extensive marsh upon its southern and western border, through which flows Gunner's Run, and from the Delaware on the east. Dr. Edgar Janvier, of Richmond, says:—'We are scarcely ever withont cases of intermittent and remittent fever, though fewer cases have occurred within the last twelve months than previously. During the autumn of 1850 they prevailed to a considerable extent on our river road as far as Bridesburg, but this spring, throughout our neighborhood, there is an exemption.' Dr. J. reports a few epidemic cases of typhoid fever in Richmond during the year 1850. Pertussis, rubeola, scarlatina, and varicella have prevailed epidemically at different periods during the year, but have manifested nothing unusual in their type. The scarlet fever epidemic Dr. Janvier believes to have been peculiarly severe and fatal in this district. He saw some cases die within 24 or 48 hours of the attack. The disease did not appear to be influenced as much by intercourse as by the epidemic constitution of the atmosphere. Many families enjoyed entire exemption, while severe and fatal cases occurred in the same block.

Physicians practising in Richmond district state that the coal laborers are unusually healthy, notwithstanding the large quantity of fine coal dust swallowed and inhaled by them, and which forms a complete coating to the skin. The appetite of the laborers is excellent, and delicate persons are said often to become strong and vigorous while working among the coal. So completely does the fine dust penetrate the tissues, that not only the expectoration is loaded with it, but the discharges also; and yet dyspepsia and pulmonary consumption are rare among the laborers. There would seem, indeed, to be a decidedly healthful influence exerted by the coal dust upon those who are constantly exposed to it; a fact which may suggest an important hint to practising physicians.

KENSINGTON.—Dr. Helffenstein writes to the committee that measles and scarlet fever have prevailed as epidemics in 1850; most of the cases of the latter disease were of a mild character, the most severe and fatal occurring in the latter part of the year. Intermittent and remittent fevers are common in this district, though they were less prevalent in 1850 than in some former years. Abscesses, felons, and carbuncles have prevailed in Kensington to an unusual extent, and amongst all classes of people.

WEST PHILADELPHIA.—This section of the county appears, from the report of Dr. Gallagher, of Mantua, to have suffered less from epidemic disorders than many other portions. Dr. G. remarks: "I have seen very few cases of scarlatina, altogether mild in its form, and yielding very readily to ordinary treatment." Malarial fevers prevail in this district, especially along the banks of the Schuylkill to a greater or less extent every year. In the year 1850 these diseases were not so prevalent as usual. Typhoid fever, according to Dr. Gallagher, has prevailed more or less in this district for the past sixteen years; the cases usually occur in the autumn, and Dr. Gallagher has observed that the later in the season the more severe and fatal is the disease. There appears to have been less sickness, and that of a milder form, in this district, in 1850, than for years past.

SPRING GARDEN AND PENN DISTRICT.—The report from this part of the city, which is from Dr. Wilson Jewell, is somewhat lengthy and well considered. "As an evidence of the comparative immunity of these districts from diseases epidemic in their character, we have only to advert to the statistics of the cholera in 1849, as published by the Board of Health, where it is stated that while in Southwark, Moyamensing, and Richmond, the ratio of cases of cholera to population was as one to every 136 inhabitants, Penn and Spring Garden presented but one in every 514 inhabitants. Spring Garden and Penn suffered from scarlet fever in the commencement of 1850, but to a less degree than some other sections of the county. In the northwestern extremities of Spring Garden and Penn districts, as well as in some other unimproved portions of the districts, malarious fevers of an intermittent and remittent type prevail endemically during the summer and autumn months, caused by miasmatic exhalations arising from decomposing animal and vegetable substances exposed to the rays of the sun, and which are at all times fruitful cases of disease and active agents in the propagation of fevers. The ponds of stagnant water on unimproved lots and in the vicinity of brickyards are also cited as among the causes, beside the frequent turning up of the soil at brickyards, the levelling of lots, filling up and digging down new streets, digging of cellars and filling up of lots which lay below the level of streets."

SOUTHWARK.—"The only low and wet part of the district is small in extent, situated on the bank of the river Delaware to the south and east. It formed originally the commencement of the alluvial meadows, which extend along the whole of the western shore of the river to the mouth of the Schuylkill. The banks to the southern line of the district are now in ruins, and the meadow land is constantly overflowed. The principal streets of the built portion of the district are well paved and drained. Most of the drainage is on the surface, the extent of culverts within the district being very limited. The streets are kept perfectly clean, and there is no accumulations of filth in any part of the built portion of the district. The houses are mostly of modern construction, and though frequently small in size, are tolerably well ventilated. All are plentifully supplied with the Schuylkill water. We allude now to the houses fronting on the main streets. Unfortunately, the cupidity of capitalists has induced them to construct numerous courts and alleys. In some of those sufficient ventilation is not provided for, and the houses are crowded with a miserable, improvident population, by whom little attention is paid to cleanliness and domestic comfort. In general, however, the population of Southwark is composed of industrious, thrifty mechanics, and their families. Having the means to command the comforts of life, they feel a pride in the neatness and cleanliness of their houses and persons. The pauper and abandoned population of the district is comparatively small. It is sufficiently extensive, however, to crowd the small houses in the narrow courts and alleys, and to occasion no small amount of disease and suffering, espectially during the winter seasons." The report from which the above is extracted, which is by Dr. D. Francis Condie, remarks that during the past year no serious epidemic has prevailed. Towards the close of the summer and the principal part of the autumn, remittent fevers prevailed to a very considerable extent south of the compactly built portion of the district, and along the whole of its river front. It was protracted in duration, though not particularly severe, and seldom fatal.

*Friday*, 31*st May*.—Dr. Samuel Jackson, late of Northumberland, offered the following resolution, which was adopted after an interesting discussion:

Resolved, That this Society approves the recommendation of the Philadelphia County Medical Society in favor of a change in the mode of representation of the National Medical Association, making such representation to consist exclusively of delegates from State and County Medical Societies.

Much of the session was occupied in the reading of reports of the medical topography of Berks, Chester, Blair, Montgomery, Delaware, and Huntingdon counties. The report from Berks was voluminous, and excited general remark from its completeness.

These reports contained accounts of the epidemic diseases of the several counties from which they came, with interesting sketches of their medical topography, prepared by Committees of these Societies. It appears from these reports, that a severe epidemic dysentery had prevailed within the past year in Chester county, an excellent account of which is reported by Dr. Garton, of that county. Intermittent and remittent fevers had prevailed in various marshy districts, while on high and dry lands no such diseases had appeared.

Scarlet fever appears to have prevailed in all the counties from which reports were received.

From Montgomery county, a report prepared by Dr. Corson, gives an account of a terrific epidemic erysipelas, which appeared in the high lands about two miles east of Norristown in the fall of 1847. At the same time a puerperal fever occurred amongst the women of this district.

Dr. West, from a Committee appointed at the last annual session to consider the subject of establishing a pathological museum in connection with the State Society, reported that the project, although desirable, would require a large fund to effect it, which the Society does not possess. They report however, that the Philadelphia College of Physicians is engaged in an effort of the same kind, and they therefore recommend that any member of the State or County Societies having pathological specimens, which they are willing to contribute towards the formation of such a museum, be requested to forward them to the College of Physicians who will take charge of them as a nucleus until the State Society is ready to make its own effort, and throw open the collection to the State Society for inspection. The report was adopted.

The question of the next annual session was then discussed. The general sentiment seemed to be in favor of Philadelphia as the place of meeting, and it was accordingly fixed upon. The time agreed upon was the last Wednesday in May.

The committee on nominations reported the following list of nominations for officers of the Society for the ensuing year.

*President.*—Dr. Charles Innes, of Northampton.

*Vice Presidents.*—Dr. Joseph B. Ard, of Mifflin; John Baskin of Mercer; George W. Norris, of Philadelphia; John P. Heister, of Reading.

*Recording Secretaries.*—Drs. Isaac Remington, of Philadelphia, and C. D. Gloninger, of Lebanon.

*Corresponding Secretary.*—Dr. Isaac Hays, of Philadelphia.

*Censors.*—1st and 2d Districts—Dr. Geo. B. Wood, of Philadelphia; Geo. B. Kerfoot, of Lancaster; Isaac Thomas, of Chester county; Jesse Young, of Delaware; John W. Gloninger, of Lebanon. 3d and 4th Districts—Drs. John D. Ross, of Blair; J. H. Case, of Perry; W. McIlvaine, of York; Joseph Henderson, of Mifflin, Joseph B. Ard, of Mifflin. 5th and 6th Districts—Drs. J. P. Gazzam, of Allegheny; John Baskin, of Mercer; C. Perkins, of Erie; W. Addison, of Allegheny; G. D. Bruce, of Allegheny.

*Delegates to the National Medical Convention for* 1852.—Drs. Isaac R. Walker, of Chester; Traill Green, of Northampton; George W. Norris, of Philadelphia; Elwood Harvey, of Delaware; F. S. Burrows, of Lancaster; John L. Foulke, of Montgomery; J. P. Heister, of Berks.

Dr. Ard declined the nomination as one of the censors of the 3rd and 4th districts, as he had removed from Mifflin county to Philadelphia; and, on his motion, Dr. G. B. Mitchell was substituted in his place; the report was then adopted, and the persons nominated elected to their several offices.

An interesting report was received from Professor Jackson, on the subject of small-pox. This report was from a committee appointed at a previous annual session, and a report was made at the session of 1850 on the subject, the committee being continued from year to year. The investigations of the committee develop some very important facts.

Dr. Emerson offered the following resolution which was adopted:

Resolved, That a Special Committee of the Medical Society of the State of Pennsylvania, be appointed to investigate the accuracy of the conclusions put forth by Drs. Gregory, of London, and Cazenave of Paris, in relation to Small-pox and Vaccination, as referred to in the report made to this Society by the Committee on Small-pox and Vaccination.

The Chair appointed Dr. Emerson, Prof. Jackson, Drs. Warrington, Parrish, and John D. Griscom.

On motion of Dr. Emerson, a committee of three was appointed to memorialize the Legislature in favor of the gratuitous revaccination of the poor throughout the State. Drs. Emerson, Condie, and Jewell, were appointed the committee. On motion of Dr. Condie, the co-operation of the county societies and the profession generally throughout the State, was requested.

The thanks of the Society were tendered to Dr. Worthington for his able and efficient services as President, to which he briefly replied, and yielded the chair to his successor, Dr. Innes. The latter gentleman delivered a few words of introductory address, which were well received.

The Society then adjourned to meet on the third Wednesday of May, 1852, in this city.—*N. Amer. and U. S. Gaz.*

